# Evidence of Multifractality from Emerging European Stock Markets

**DOI:** 10.1371/journal.pone.0040693

**Published:** 2012-07-17

**Authors:** Petre Caraiani

**Affiliations:** Institute for Economic Forecasting, Romanian Academy, Bucharest, Romania; Universidad Veracruzana, Mexico

## Abstract

We test for the presence of multifractality in the daily returns of the three most important stock market indices from Central and Eastern Europe, Czech PX, Hungarian BUX and Polish WIG using the Empirical Mode Decomposition based Multifractal Detrended Fluctuation Analysis. We found that the global Hurst coefficient varies with the q coefficient and that there is multifractality evidenced through the multifractal spectrum. The exercise is replicated for the sample around the high volatility period corresponding to the last global financial crisis. Although no direct link has been found between the crisis and the multifractal spectrum, the crisis was found to influence the overall shape as quantified through the norm of the multifractal spectrum.

## Introduction

There is a long interest in modeling financial markets that span well beyond the disciplines of finance and economics attracting mathematicians, physicists and many others from different fields. The attractiveness of financial markets comes not only from its complex dynamics that result from the interactions of a multitude of agents but also from its presence and influence in our daily life as the last financial crisis has proved it. One of the questions that emerged in the last decades was whether financial markets are characterized by chaos and fractality.

Before going further, we clarify a few key concepts for the general audience. By efficient financial market we understand, following [1], a market where prices reflect in a full manner all the information available and, moreover, they adjust in a quick manner when new information becomes available. We also use the concept of daily (index) returns by which in this paper we understand the logarithmic difference of a stock market index between its closing price in a certain day and its closing price a day earlier.

The discipline of economics has not remained indifferent to the rapid emerging field of fractal and chaos theory. The development of testing techniques in the fields of mathematics and physics has started to be felt in economics in the early `80’s when early tests for the presence of fractal dimension and chaotic behavior in economic and financial processes were applied, see [2] and [3] for a review of early results. Until now, the idea of chaos and fractal behavior remains debatable in the field of economics and finance, mostly due to the specific of economic time series characterized by relatively short samples (the accurate computations of correlation dimension or the maximum Lyapunov exponent require large samples) and the presence of noise. [4] summarized the research taken in the `80’s and `90’s by pointing that there is no evidence of “within the structure of the economic system” as current tests cannot determine the source of detected chaos.

At the same time, as some of the research points out, [3] and [4], there is a further need to further develop tests and deepen the topic of chaos and fractality in the field of economics. The need is even more urgent in the discipline of finance. The still dominant paradigm of efficient stock markets as outlined by [1] has serious weaknesses, among which we can enumerate time dependent self similarity, see [5] and [6] for a larger review. Such weaknesses called for alternative theories, one of which is worth mentioning in the context of present paper, namely the fractal market hypothesis due to [6]. According to [5], the fractal market hypothesis assumes that asset returns are dependent on both frequency and time horizon and that there is global dependency manifested through its fractality. This hypothesis has been reinforced by the discovering of multifractals in the asset returns, see [7] for early findings, which develops earlier ideas by [8] as well as [9].

Although there is a growing work on multifractality for either developed stock markets, see [7], or emerging stock markets, [10] or [11] , the literature not only on multifractality, but in general in testing for chaotic and fractals behavior in CEE stock markets is very limited. Nevertheless, some papers are worth mentioning. [12], using a Hurst coefficient derived on the basis of the wavelet decomposition, found evidence for long run dependence on some of the CEE stock markets. They also found evidence of a time dependent value for the Hurst coefficient. In a recent paper, [13], using the Hurst coefficient determined on the basis of the Detrended Fluctuation Analysis, analyzed the dynamics of daily returns of share prices of 126 selected companies from the Warsaw Stock Exchange. He found that the after the drop in the Hurst exponent, the change in either long-term trend or in the long-term rate of return has an increased probability than for points randomly selected from the whole sample.

This paper proposes itself to answer to several questions, namely whether the daily returns in the selected CEE stock market indices are characterized by multifractality, how much using a surrogate data series, shuffled ones, leads to changes in the results. Not at last, we also investigate whether the crisis period has lead to different strenghts of multifractal spectrum, as suggested in an earlier work on the 1987 financial crisis by [14].

The paper is organized as follows. The methodology used throughout the paper is explained in the second section. The third section presents the empirical results and discusses the results. The last section draws the conclusions and outlines some possible extensions of this paper.

## Methods

The methodology is based on the Empirical Mode Decomposition, EMD hereafter, based Multifractal Detrended Fluctuation Analysis, (EMD based MFDFA hereafter). There are a number of techniques to derive the multifractal spectrum of a time series, some based on wavelets, other based on detrended fluctuation analysis.

Basically, EMD based MFDFA is a development of the now well established technique of Multifractal Detrended Fluctuation Analysis, MFDFA hereafter, due to [15]. We discuss first the EMD approach in decomposing time series, and present then in a comparative way the standard MFDFA as well as the EMD variation.

**Figure 1:**
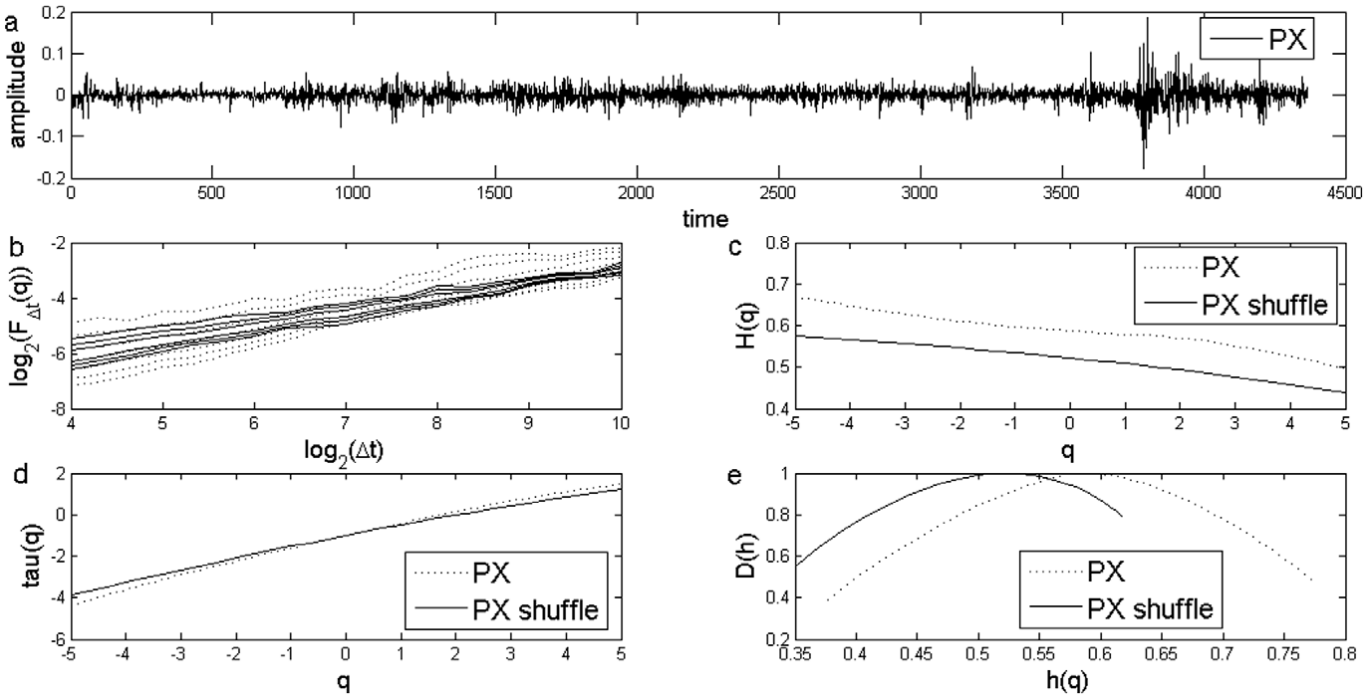
Standard MF-DFA Analysis of Czech Stock Market Index PX. a) Daily returns for Czech Stock Market Index PX; b) Log Scaling Function; c) q-generalized Hurst Exponent; d) Multifractal spectral scaling exponent τ(q) versus q; e) Multifractal spectrum.

**Figure 2:**
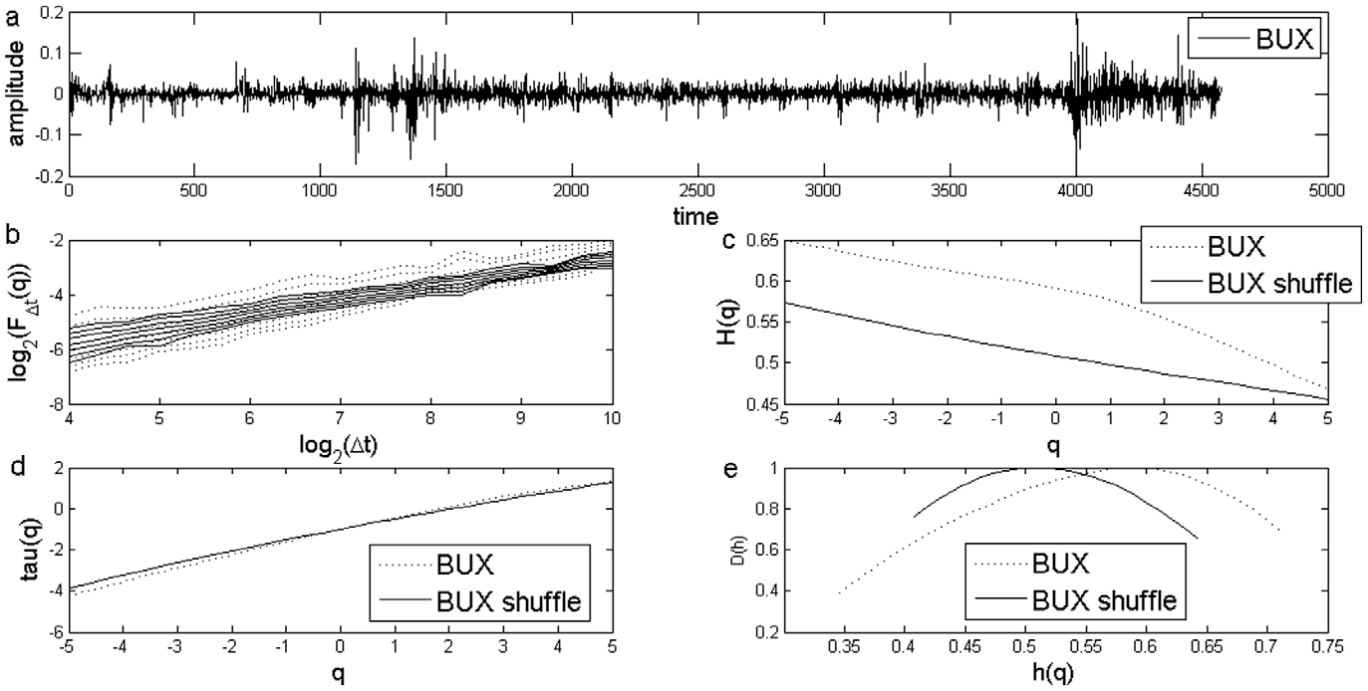
Standard MF-DFA Analysis of Hungarian Stock Market Index BUX. a) Daily returns for Hungarian Stock Market Index BUX; b) Log Scaling Function; c) q-generalized Hurst Exponent; d) Multifractal spectral scaling exponent τ(q) versus q; e) Multifractal spectrum.

**Figure 3:**
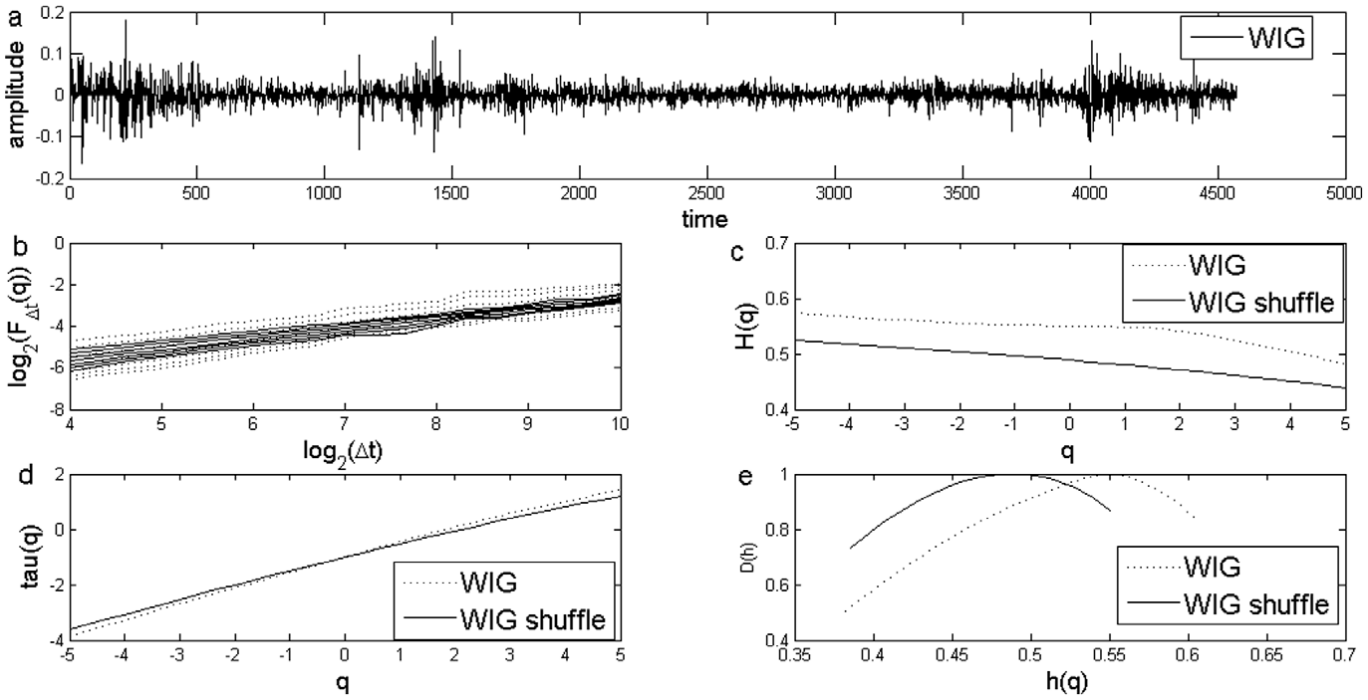
Standard MF-DFA Analysis of Polish Stock Market Index WIG. a) Daily returns for Polish Stock Market Index WIG; b) Log Scaling Function; c) q-generalized Hurst Exponent; d) Multifractal spectral scaling exponent τ(q) versus q; e) Multifractal spectrum.

### The Empirical Mode Decomposition

The Empirical Mode Decomposition, is a new technique in signal theory due to [16]. Several papers have outlined its advantages with respect to other filtering techniques, see) [17] or [18]. As [16] showed, essentially, the EMD consists in decomposing a certain time series into a finite number of so-called intrinsic mode functions. These functions have to fulfill two essential conditions. The first one says that the numbers of local extreme and the numbers of zero crossings, for the entire sample of data, must be equal or differ by 1 at most. The second condition states that at any point in time, the mean value of the “upper envelope”, as given by the local maxima, and the “lower envelope”, given by the local minima, must be zero.

**Table 1:** Multifractal strength for standard MFDFA.

Series	Multifractal strength	
	Initial series	Shuffled Series
Czech PX	0.69	0.44
Hungarian BUX	0.54	0.34
Polish WIG	0.44	0.50

Source: Own computations.

When one compares it the wavelet approach or the Fourier approach, one notices that it enjoys several advantages. Compared with the Fourier approach, it gives a representation in both time and frequency and it also allows working with nonstationary data while compared with wavelets it also can work with nonlinear time series. We detail the algorithm below:

1. For a given time series *y(t)*, one identifies all extrema;
2. Using an interpolation procedure, the local maxima result in an upper envelope *U(y)*;
3. In a similar manner, from the minima, a lower envelope results, *L(y)*;
4. One derives the mean envelope as:

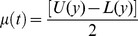

1. This mean is extracted from the signal, so that a new series results:

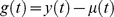

1. Finally, one verifies whether the new series *g(t)* satisfies the two above mentioned conditions.

If the conditions are met, the algorithm is stopped, if they are not, the algorithm continues. In the end, the trend is given by:
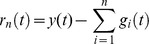
Where 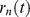 represents the trend of the series.

### The MFDFA Based on Empirical Mode Decomposition

The introduction of EMD based MFDFA can be traced back to [19]. The development assumes that the first two steps of MFDFA remain the same. In the third step, instead of a polynomial detrending, specific to detrended fluctuation analysis, the EMD is used to decompose the series. The method used in this paper is described below, following [15] and [19].

**Figure 4:**
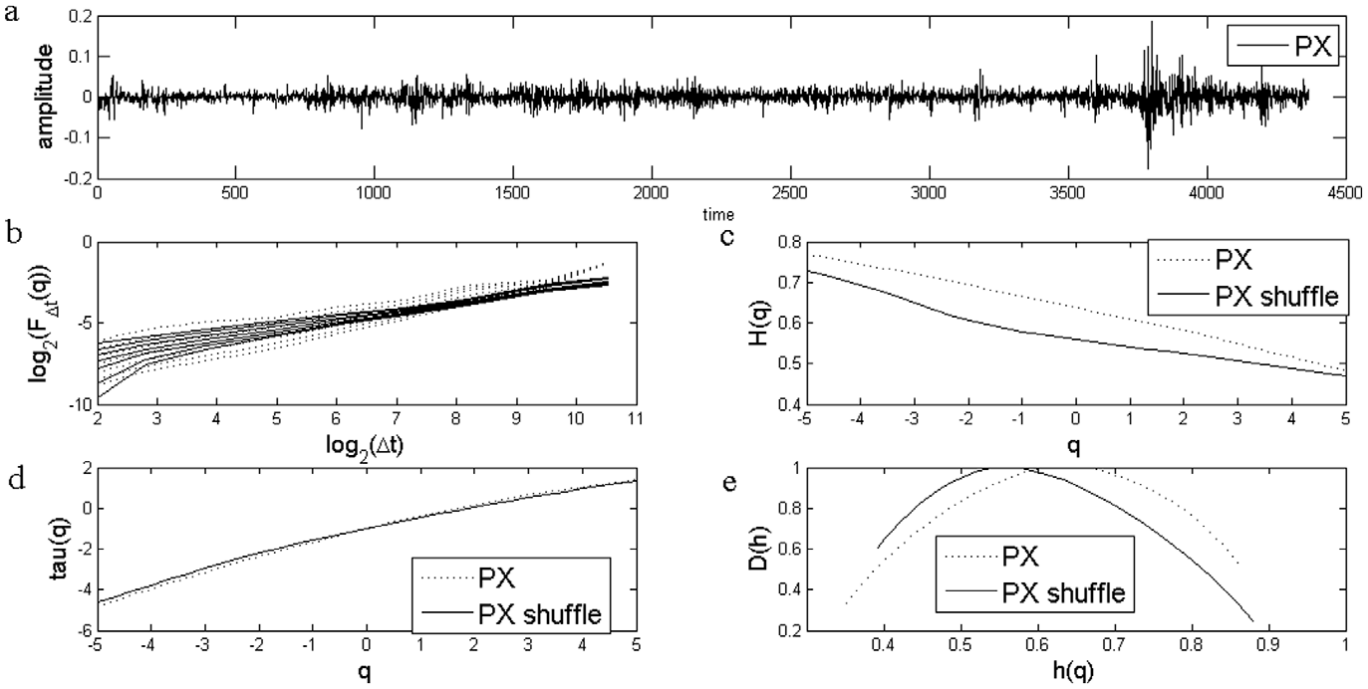
EMD based MF-DFA Analysis of Czech Stock Market Index PX. a) Daily returns for Czech Stock Market Index PX; b) Log Scaling Function; c) q-generalized Hurst Exponent; d) Multifractal spectral scaling exponent τ(q) versus q; e) Multifractal spectrum.

**Figure 5:**
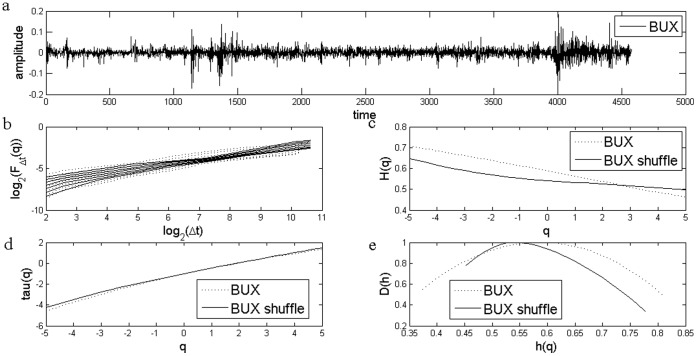
EMD based MF-DFA Analysis of Hungarian Stock Market Index BUX. a) Daily returns for Hungarian Stock Market Index BUX; b) Log Scaling Function; c) q-generalized Hurst Exponent; d) Multifractal spectral scaling exponent τ(q) versus q; e) Multifractal spectrum.

**Figure 6:**
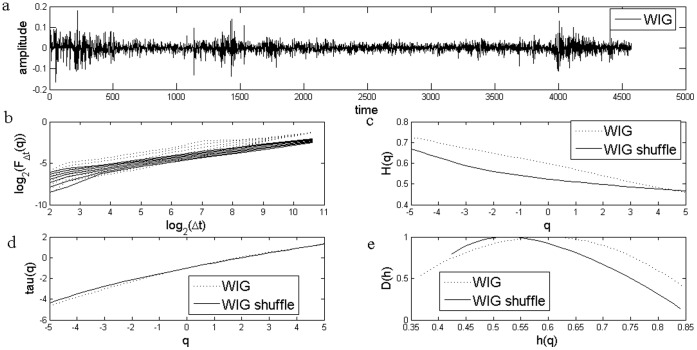
EMD based MF-DFA Analysis of Polish Stock Market Index WIG. a) Daily returns for Polish Stock Market Index WIG; b) Log Scaling Function; c) q-generalized Hurst Exponent; d) Multifractal spectral scaling exponent τ(q) versus q; e) Multifractal spectrum.

We start from a given time series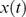 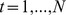. In the first step we derive a profile of the series which is nothing more than a cumulative sum:
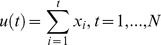
(1)


Next, in the following step, we partition the profile ,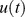, in segments each one of equal size *s*, with the property of being disjoint, where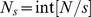. Here 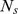
is determined as the ratio between *N* and the scale factor *s*.

Each of the segments 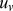 has the following property:
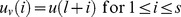
(2)


Here *l* is determined from: 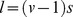.

The step three of the algorithm in the baseline MFDFA implies the detrending of the segments using a polynomial fitting. In the version based on the empirical mode decomposition, one computes an EMD local trend for each segment 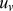 as 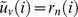, where 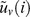
is the local trend and 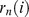 is local trend based on the EMD approach, see the previous section.

One constructs then the series of residuals using the trend function as follows:
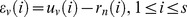
(3)


Using the residuals determined in equation (3), the detrended fluctuation function 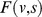 for a segment 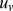 is given by:
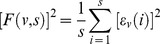
(4)


Based on this we derive the q-th order overall detrended fluctuation function as:
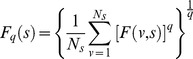
(5)


Where *q* can take any real value except q = 0. In case q = 0, the formula becomes:
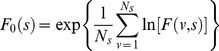
(6)


Finally, based on different timescales s, a power-law relationship can be established between 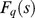 and the time scale *s*:
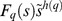
(7)


Here 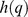 stands for the generalized Hurst index.

Further, for a each *q* a corresponding function 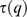 can be determined by:
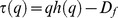
(8)


With 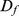 representing the multifractal spectrum.

**Table 2:** Multifractal strength for EMD based MFDFA.

Series	Multifractal strength	
	Initial series	Shuffled Series
Czech PX	0.67	0.47
Hungarian BUX	0.49	0.65
Polish WIG	0.58	0.86

Source: Own computations.

## Results

### Data Used

The data consist in daily returns of main stock market indices in Czech Republic, Hungary and Poland. All the data were taken from DataStream. The data were transformed, as usual in the literature, in US dollar denominated values. Before applying the statistical techniques, the price indices were transformed in log-returns.

**Figure 7:**
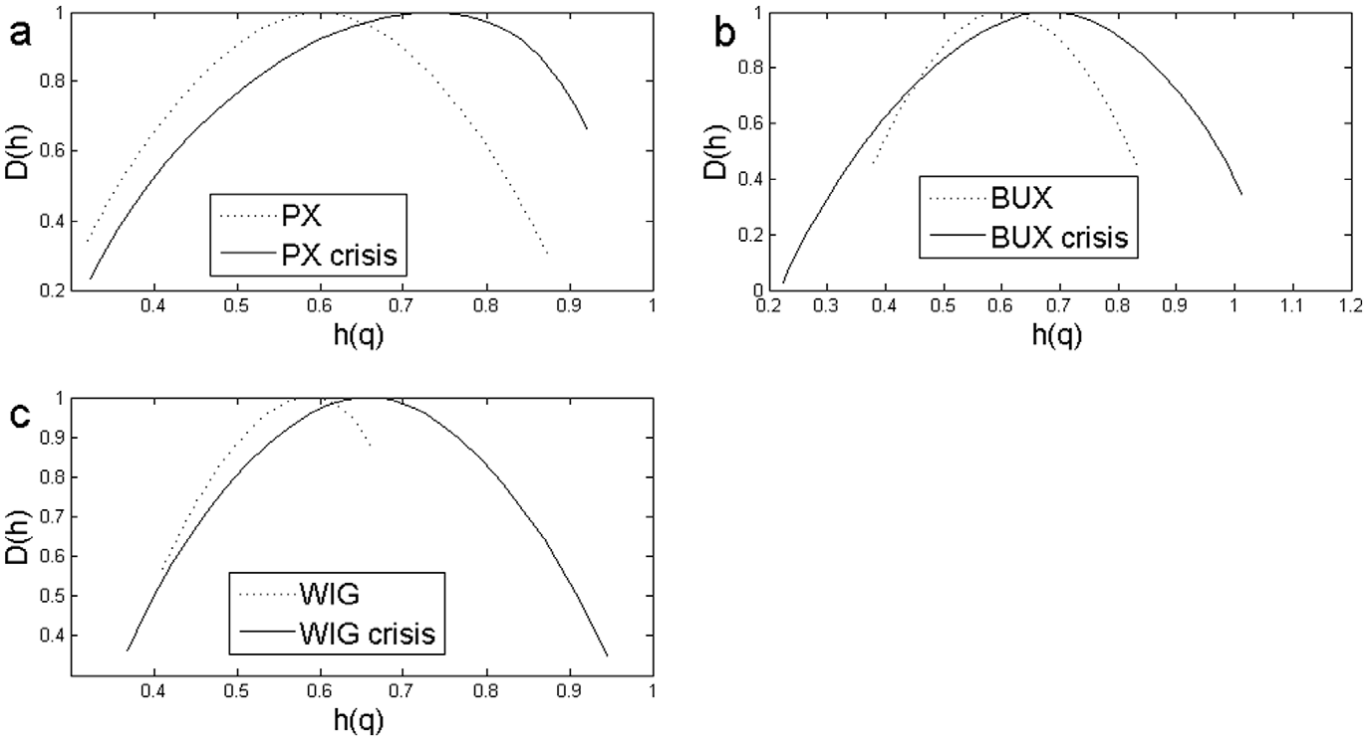
The Impact of the Crisis Analyzed Using Standard MF-DFA. Multifractal spectrum for the whole sample compared with 2008–2009 sample that includes the high volatility period using standard MFDFA: a) Czech case: PX for the whole sample and PX crisis for 2008–2009 period; b) Hungarian case:BUX for the whole sample and BUX crisis for 2008–2009 period; c) WIG for the whole sample and WIG crisis for a 2008–2009 subsample.

**Figure 8:**
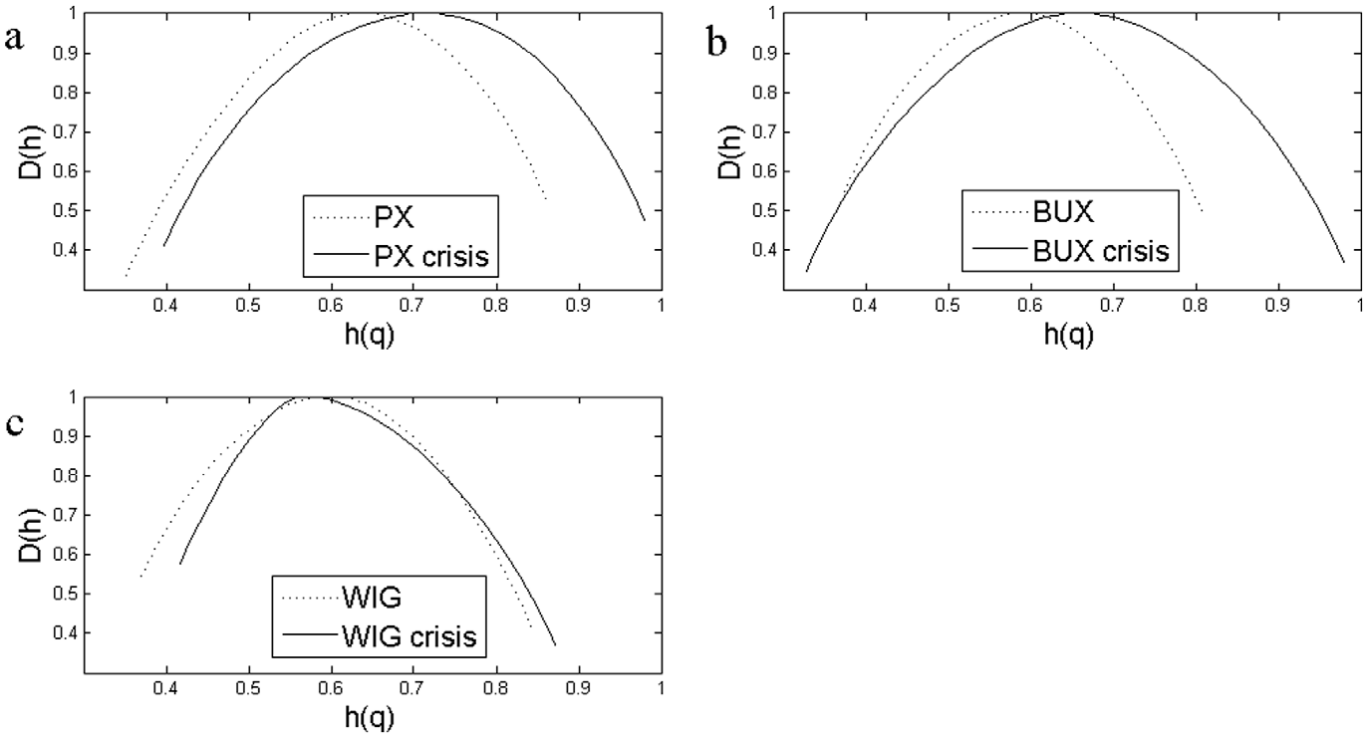
The Impact of the Crisis Analyzed Using EMD based MF-DFA. Multifractal spectrum for the whole sample compared with 2008–2009 sample that includes the high volatility period using EMD based MFDFA: a) Czech case: PX for the whole sample and PX crisis for 2008–2009 period; b) Hungarian case:BUX for the whole sample and BUX crisis for 2008–2009 period; c) WIG for the whole sample and WIG crisis for a 2008–2009 subsample.

The data for Czech Republic consists in daily observations for PX index from April 1994 to December 2010. Overall, 4369 observations are used. For Hungary, we used daily data on BUX index, dating from June 1993 to December 2010, with a total number of observations of 4577. The last index, the one for Poland, consists in daily observation for the WIG index, dated between June 1993 and December 2010, overall 4575 observations being used.

### Standard MF-DFA

The results for the standard MFDFA for the three stock market indices are presented in Figures 1, 2 and 3. The procedures used an upper bound for *q* of 5, a lower bound of −5 and considered 31 elements in the vector *q*. For each case, results for the case of the shuffled time series are also presented. Shuffled time series are obtained from the original series after eliminating the serial correlation.

**Table 3:** Multifractal strength in crisis compared to full sample using standard MFDFA.

Series	Multifractal strength	
	Full sample	Crisis sample
Czech PX	0.69	0.76
Hungarian BUX	0.54	0.97
Polish WIG	0.44	0.65

Source: Own computations.

**Table 4:** Multifractal strength in crisis compared to full sample using EMD based MFDFA.

Series	Multifractal strength	
	Full sample	Crisis sample
Czech PX	0.67	0.58
Hungarian BUX	0.49	0.65
Polish WIG	0.58	0.62

Source: Own computations.

The Hurst coefficient for each series, as shown in the literature, is given by H(q) for q equal to 2. I obtained a Hurst coefficient for Czech PX of 0.57, for Hungarian BUX of 0.55, while for the last case of Polish WIG, Hurst coefficient was estimated at 0.54. These estimations indicate persistence of the time series and are usually interpreted as an indicator of an emerging financial market, see [10] and they are consistent with the results from other studies, see [13].

First of all, there is evidence of multifractality from the dependence of the *H(q)* from the *q* moment, as presented in Figures 1c, 2c and 3c. Moreover, there is a decreasing trend for *H(q)* which is a clear sign of multifractality as indicated by the literature. In order to characterize the multifractality, the multifractal spectra are presented in Figures 1e, 2e and 3e.

**Table 5:** Norms of the multifractal spectra in crisis compared to full sample based on MFDFA.

Series	Norm of the multifractal spectrum	
	Full sample	Crisis sample
Czech PX	5.36	5.71
Hungarian BUX	5.57	5.29
Polish WIG	5.82	5.51

Source: Own computations.

**Table 6:** Norms of the multifractal spectra in crisis compared to full sample using EMD based MFDFA.

Series	Norm of the multifractal spectrum	
	Full sample	Crisis sample
Czech PX	5.55	5.72
Hungarian BUX	5.53	5.44
Polish WIG	5.48	5.55

Source: Own computations.

There are some variations with respect to the amplitude of the fractal spectrum, given by the formula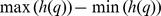, see Table 1. We would like to know whether the shuffled series have a different multifractal strength. We apply the χ^2^-test for association with which we can test whether there is any influence of shuffling the series on the multifractal strength. When running the test we get there is no influence of shuffling the series (p-value is of 0.47).

### EMD Based MF-DFA

We follow the same procedure in applying the EMD version of the MFDFA, using values for *q* between −5 and 5 and 31 elements for *q*. We also apply the procedure for the shuffled series. The results are presented in Figures 4, 5 and 6.

We look again at the results for the Hurst coefficient which are given by H(q) for q equal to 2. We obtained similar results, namely a Hurst coefficient for Czech PX of 0.57, for Hungarian BUX of 0.53, while for the last case of Polish WIG, Hurst coefficient was estimated at 0.53.

We also present the multifractal strength for each case including the shuffled series, Table 2. We test again if shuffling the series led to changes in the strength of the multifractal series. The χ^2^-test for association is used and the results indicate as in the standard case that shuffling the series did not modify the multifractal strength (p-value is of 0.39).

### The Impact of the Crisis

Another question that I answer to in this paper is whether the global financial crisis has led to increased multifractality in the selected stock markets. As showed by [14], the financial crisis from 1987 led to changes in the diameter of the multifractal spectra, signaling an increased complexity in financial data. We discuss in this section whether a similar phenomenon occurred in the emerging financial markets from Europe. Again we apply both approaches in deriving the multifractal spectra of the time series in cause.

Figure 7 and 8 shows the multifractal spectra computed for the whole period as well as for a subsample corresponding to the financial crisis period. We computed the multifractal spectrum for a subsample of two years, January 2008 to December 2009, roughly corresponding to the crisis period, also Figures 1a, 2a and 3a. Two entire years were selected as the precise date when the crisis spilled to a particular financial market is hard to determine.

### Is the Multifractal Strength Different during the Crisis?

The multifractal strengths are presented in Tables 3 and 4. When testing for any influence of the crisis on the multifractality using the χ^2^-test for association we cannot find any statistical influence of the crisis on the multifractality of the series as synthesized in the multifractal strength (the p-values are of 0.35 for the standard MFDFA and of 0.40 for the EMD version).

### Has the Multifractality Changed during the Crisis?

We discuss here further evidence regarding the shape and distribution of the multifractal spectrum for the selected emerging European stock markets. While in the previous section we focused on the multifractal strengths, here we take a look at the multifractal spectra taken as a whole. While the approach in the previous section was justified on the grounds that most of the research on the multifractality of the financial time series has been interested first of all in the multifractal strength of the series. However, given the fact that the multifractal strength of a time series has not only a maximum but also width and a parabolic distribution, we quantify each of the multifractal strengths through a Euclidean norm.

We use the p-norm, the 2-norm to be more precise, to characterize the multifractal spectrum of the series given the fact that the multifractal spectrum is a line in a two dimensional space. The 2-norm is also known as Hilbert – Schmidt norm and it is given as:
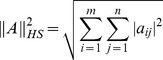



We compare the norms of the multifractal spectra for each series for the whole sample as well as for the crisis period.

The results are presented in Table 5 and 6. We apply again the χ^2^-test for association to see if there is any influence from the crisis on the multifractal spectra. In this case the values of the computed χ^2^-test (0.000245 for the baseline MFDFA approach as well as 0.000244 for the EMD based MFDFA) indicate that the crisis has clearly influenced the overall shape of the multifractal spectrum.

## Discussion

The accumulation of evidence in the favor of chaotic patterns, fractality and multifractality in economic and financial time series is an important contribution in the understanding of the complexity of economic and financial processes. In this paper, we add to the existing evidence on multifractality in financial time series by using daily returns from three of the key stock market indices in Central and Eastern Europe.

We showed that the global Hurst coefficient varies with the moment q, and that the series are characterized by a multifractal spectrum. We compared the results from the initial time series with those obtained on the basis of shuffling the time series which we found that did not influence the results. We also studied the impact of the financial crisis the multifractal spectrum for the overall periods for each stock market index with those for a subsample of two years, 2008 to 2009, the years of the last big financial crisis. The overall evidences found here, although not a clear argument in the favor of an increased multifractal strength, point to a more complex change in the shape of the multifractal spectrum.

Further studies could deepen the topic by analyzing the factors that drive the strength of the multifractal spectrum, its relationship to the degree of financial development or its behavior during the periods of financial crisis.
